# Supplementary material for: Percutaneous nephrostomy guidance by a convolutional-neural-network-based optical coherence tomography endoscope
Source: Commun Eng. 2026 Mar 6;5:47. doi: 10.1038/s44172-026-00613-8 (PMC12976127; doi:10.1038/s44172-026-00613-8)
Supplement: Supplementary file 2 — Percutaneous Nephrostomy Guidance by a Convolutional-Neural-Network-Based Optical Coherence Tomography Endoscope: Supplementary Document [file 44172_2026_613_MOESM2_ESM.pdf]

Percutaneous Nephrostomy Guidance by a Convolutional-Neural-Network-Based Optical Coherence Tomography Endoscope: Supplementary Document

TABLE S1: Confusion matrices of renal tissue recognition using ultrasound by two radiologists.

Radiologist 1

**Cortex**		Prediction			**Medulla**		Prediction			**Calyx**		Prediction	
		Pos	Neg				Pos	Neg				Pos	Neg
Truth	Pos	2	14		Truth	Pos	4	9		Truth	Pos	3	7
	Neg	5	29			Neg	10	27			Neg	9	31

**Sinus fat**		Prediction			**Pelvis**		Prediction	
		Pos	Neg				Pos	Neg
Truth	Pos	5	2		Truth	Pos	0	4
	Neg	9	34			Neg	2	44

Overall recognition rate = 28% (14/50)

Radiologist 2

**Cortex**		Prediction			**Medulla**		Prediction			**Calyx**		Prediction	
		Pos	Neg				Pos	Neg				Pos	Neg
Truth	Pos	3	13		Truth	Pos	5	8		Truth	Pos	4	6
	Neg	5	29			Neg	11	26			Neg	9	31

**Sinus fat**		Prediction			**Pelvis**		Prediction	
		Pos	Neg				Pos	Neg
Truth	Pos	3	4		Truth	Pos	0	4
	Neg	9	34			Neg	1	45

Overall recognition rate = 30% (15/50)

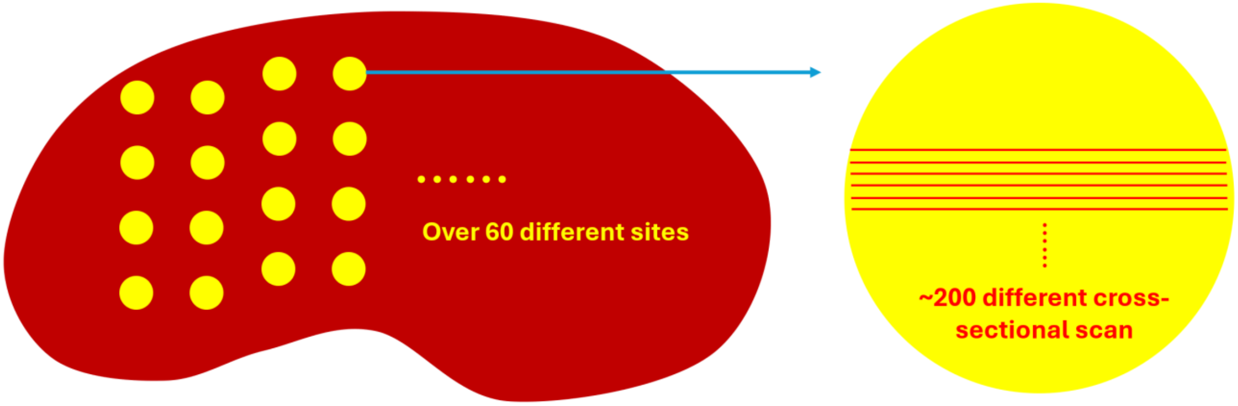

FIGURE S1: Cross-sectional scanning schematic method.

To help evaluate robustness across subjects, here we provide per-kidney confusion matrices and per-kidney accuracy/recall/precision for the internal test set with 5 kidneys (with 95% confidence intervals). The matrices for the classification internal results are shown below.

TABLE S2: matrices for the classification internal results.

Internal kidney - 1

		Predicted Cortex	Predicted Medulla	Predicted Calyx	Predicted Fat	Predicted Pelvis
Truth	Cortex	10000	0	0	0	0
Truth	Medulla	0	10000	0	0	0
Truth	Calyx	0	0	10000	0	0
Truth	Fat	0	0	0	10000	0
Truth	Pelvis	0	0	0	0	10000

Internal kidney - 2

		Predicted Cortex	Predicted Medulla	Predicted Calyx	Predicted Fat	Predicted Pelvis
Truth	Cortex	9947	0	53	0	0
Truth	Medulla	0	10000	0	0	0
Truth	Calyx	0	0	10000	0	0
Truth	Fat	47	0	54	9899	0
Truth	Pelvis	0	0	0	0	10000

Internal kidney - 3

		Predicted Cortex	Predicted Medulla	Predicted Calyx	Predicted Fat	Predicted Pelvis
Truth	Cortex	10000	0	0	0	0
Truth	Medulla	0	10000	0	0	0
Truth	Calyx	0	0	10000	0	0
Truth	Fat	0	0	0	10000	0
Truth	Pelvis	0	0	0	0	10000

Internal kidney - 4

		Predicted	Predicted	Predicted	Predicted	Predicted
		Cortex	Medulla	Calyx	Fat	Pelvis
Truth	Cortex	10000	0	0	0	0
Truth	Medulla	0	9990	10	0	0
Truth	Calyx	0	0	10000	0	0
Truth	Fat	0	0	0	10000	0
Truth	Pelvis	0	0	0	0	10000

Internal kidney - 5

		Predicted	Predicted	Predicted	Predicted	Predicted
		Cortex	Medulla	Calyx	Fat	Pelvis
Truth	Cortex	10000	0	0	0	0
Truth	Medulla	0	10000	0	0	0
Truth	Calyx	0	0	9999	1	0
Truth	Fat	0	0	0	10000	0
Truth	Pelvis	0	0	0	0	10000

For blind test, it can be seen in Table 5 of the manuscript.

Also, we have examined the inter-kidney classification behavior and tested if the utilized models trained on one subset can generalize consistently with others, with the results shown below:

TABLE S3: Inter-kidney classification behavior.

Per-kidney analysis

	Accuracy	CI lower	CI upper
**Internal kidney - 1**	1.0000	0.999923176725854	0.999999999999999
**Internal kidney - 2**	1.0000	0.999923176725854	0.999999999999999
**Internal kidney - 3**	1.0000	0.999923176725854	0.999999999999999
**Internal kidney - 4**	0.9998	0.999631850671226	0.999891356783937
**Internal kidney - 5**	1.0000	0.999886710298608	0.999996469500177

Precision

	calyx			cortex			fat			medulla			pelvis		
	value	lower CI	upper CI	value	lower CI	upper CI	value	lower CI	upper CI	value	lower CI	upper CI	value	lower CI	upper CI
**Internal kidney - 1**	1.0000	0.9996	1.0000	1.0000	0.9996	1.0000	1.0000	0.9996	1.0000	1.0000	0.9996	1.0000	1.0000	0.9996	1.0000
**Internal kidney - 2**	0.9894	0.9872	0.9912	0.9953	0.9938	0.9965	1.0000	0.9996	1.0000	1.0000	0.9996	1.0000	1.0000	0.9996	1.0000
**Internal kidney - 3**	1.0000	0.9996	1.0000	1.0000	0.9996	1.0000	1.0000	0.9996	1.0000	1.0000	0.9996	1.0000	1.0000	0.9996	1.0000
**Internal kidney - 4**	0.9990	0.9982	0.9995	1.0000	0.9996	1.0000	1.0000	0.9996	1.0000	1.0000	0.9996	1.0000	1.0000	0.9996	1.0000
**Internal kidney - 5**	1.0000	0.9996	1.0000	1.0000	0.9996	1.0000	0.9999	0.9994	1.0000	1.0000	0.9996	1.0000	1.0000	0.9996	1.0000

Recall

	calyx			cortex			fat			medulla			pelvis		
	value	lower CI	upper CI	value	lower CI	upper CI	value	lower CI	upper CI	value	lower CI	upper CI	value	lower CI	upper CI
**Internal kidney - 1**	1.0000	0.9996	1.0000	1.0000	0.9996	1.0000	1.0000	0.9996	1.0000	1.0000	0.9996	1.0000	1.0000	0.9996	1.0000
**Internal kidney - 2**	1.0000	0.9996	1.0000	0.9947	0.9931	0.9959	0.9899	0.9877	0.9917	1.0000	0.9996	1.0000	1.0000	0.9996	1.0000
**Internal kidney - 3**	1.0000	0.9996	1.0000	1.0000	0.9996	1.0000	1.0000	0.9996	1.0000	1.0000	0.9996	1.0000	1.0000	0.9996	1.0000
**Internal kidney - 4**	1.0000	0.9996	1.0000	1.0000	0.9996	1.0000	1.0000	0.9996	1.0000	0.9990	0.9982	0.9995	1.0000	0.9996	1.0000
**Internal kidney - 5**	0.9999	0.9994	1.0000	1.0000	0.9996	1.0000	1.0000	0.9996	1.0000	1.0000	0.9996	1.0000	1.0000	0.9996	1.0000
